# Polysaccharide Fraction Isolated from *Saccharina japonica* Exhibits Anti-Cancer Effects Through Immunostimulating Activities

**DOI:** 10.3390/md23010038

**Published:** 2025-01-13

**Authors:** Min Seung Park, Seung-U Son, Tae Eun Kim, Se Hyun Shim, Bong-Keun Jang, Sunyoung Park, Kwang-Soon Shin

**Affiliations:** 1 Department of Food Science and Biotechnology, Kyonggi University, Suwon 16227, Republic of Korea; alstmd0212@naver.com (M.S.P.); suson@kfri.re.kr (S.-U.S.); pillowpanda@naver.com (T.E.K.); shim505@naver.com (S.H.S.); 2 Korea Food Research Institute, Wanju-gun 55365, Republic of Korea; 3 JBKLAB, Inc., Daejeon 34013, Republic of Korea; jbk@cellmed.com (B.-K.J.); sunyoung@cellmed.com (S.P.)

**Keywords:** *Saccharina japonica*, polysaccharide, fucoidan, immunostimulating, anti-cancer, separation, sugar composition

## Abstract

The present research aimed to assess the anti-cancer effects of the polysaccharide fraction (SJP) isolated from *Saccharina japonica*. The release of immune-activating cytokines, including IL-6, IL-12, and TNF-α, was markedly stimulated by the SJP in a concentration-dependent manner within the range of 1 to 100 µg/mL. Furthermore, the prophylactic intravenous (*p.i.v.*) and per os (*p.p.o.*) injection of SJP boosted the cytolytic activity mediated by NK cells and CTLs against tumor cells. In a study involving Colon26-M3.1 carcinoma as a lung cancer model, both *p.i.v.* and *p.p.o.* exhibited significant anti-lung-cancer effects. Notably, *p.i.v.* and *p.p.o.* administration of SJP at a dose of 50 mg/kg reduced tumor colonies by 84% and 40%, respectively, compared to the control. Moreover, the anti-lung-cancer effects of SJP remained substantial, even when NK cell function was inhibited using anti-asialo-GM1. Fractionation with CaCl_2_ suggested that SJP is a mixture of alginate and fucoidan. The fucoidan fraction stimulated the immune response of macrophages more strongly than the alginate fraction. Consequently, this finding suggested that SJP from *S. japonica* possesses remarkable anti-cancer effects through the activation of various immunocytes. In addition, this finding indicates that the potent biological activity of SJP may be attributed to fucoidan.

## 1. Introduction

Algae are widely regarded as promising solutions for various diseases because of their abundant active polysaccharides, which are known to be highly functional. Approximately 100,000 varieties of algae have been identified and grouped into three classes based on their pigmentation: Ochrophyta (brown algae), Rhodophyta (red algae), and Chlorophyta (green algae) [1]. Throughout history, brown algae have played an essential role in human nutrition and diets. In particular, *Saccharina japonica* is the most commonly used species in the Republic of Korea. *S. japonica* is widely valued for its role in nutrition and traditional Eastern medicine due to its high nutritional content, which includes abundant minerals, vitamins, and non-caloric dietary fiber [2]. Brown algae, including *S. japonica*, contain various cell-wall polysaccharides which are classified into three main types: laminarin, alginate, and fucoidan [3]. Among them, alginate and fucoidan are widely utilized in the food and medical sectors because of their diverse functionalities. In summary, alginate is a polysaccharide that functions as a gelling agent, comprising guluronic acid (GulA) and mannuronic acid (ManA) linked together through β-1,4-linkages, which are irregularly repeated. Fucoidans are described in a complex manner. For example, fucoidan is extended by the α-1,3-linkage and α-1,4-linkage of fucose (Fuc) and has an irregular side chain. Furthermore, the presence of a sulfate group is the most recognizable feature of algal polysaccharides, such as fucoidan [4,5]. Their structural features have been elucidated in relative detail through various studies [1]. The influence of factors such as molecular mass, component sugar, and sugar bond type on their therapeutic effects is notable [6].

Polysaccharides isolated from various natural algae exhibit various physiological activities such as immunostimulatory, anti-colitis [7,8], anti-coagulant [9], anti-viral [10], and anti-cancer [11]. These polysaccharides have potent regulatory effects on immune responses. In particular, β-glucan derived from the brown alga *Durbilaea antarctica* suppressed colorectal cancer through the activation of the immune response of macrophages [12]. Additionally, polysaccharides isolated from *Sargassum fusiforme* stimulated immune responses, including tumor necrosis factor (TNF)-α secretion in splenocytes, thereby contributing to their anti-cancer effects [13]. The immune system is a homeostatic response that exhibits rapid and accurate aggression toward external invaders and tumor cells [14]. Tumor-cell-killing effects are substantially influenced by various immunocytes such as macrophages, natural killer (NK) cells, and cytotoxic T lymphocytes (CTLs) [15]. Macrophages are essential mediators of both natural and acquired immune systems by regulating several homeostatic and evolutionary host immune responses. Macrophages neutralize antigens through phagocytosis and digest them into a form that allows adaptive immune cells to recognize them. Furthermore, several factors, including nitric oxide (NO), interleukin (IL), and TNF, which are released from activated macrophages, contribute to various processes [16]. Moreover, the NK cell is a key cytotoxic immune cell, specifically adapted for targeting and destroying tumor cells [17]. CTLs, which are involved in adaptive immunity, exhibit strong cytolytic effects against pre-sensitized cells. In summary, various immune cells remove tumor cells through close interactions. Several studies have identified their direct toxic effects on various cancer cell types. However, studies on the effects of anti-metastatic or anti-cancer drugs through the activation of immune responses are relatively insufficient. Therefore, in the present work, we focused on confirming the immunostimulating activity of the *S. japonica*-derived polysaccharide fraction and its anti-cancer effects.

## 2. Results and Discussion

### 2.1. Activated Macrophage by Polysaccharide Fraction Isolated from S. japonica

**Figure 1:**
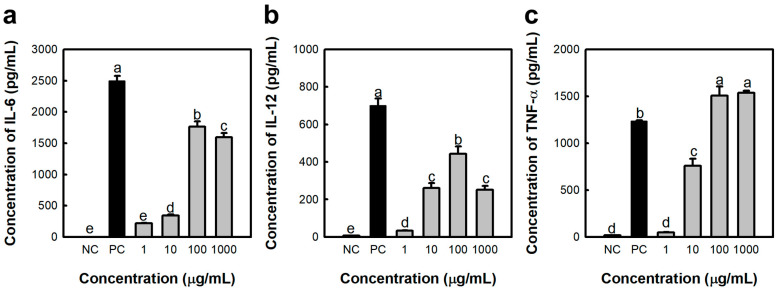
Effects of SJP isolated from *Saccharina japonica* about cytokine release by macrophages. (**a**) IL-6, (**b**) IL-12, and (**c**) TNF-α concentration of culture supernatant. The medium and LPS (1 μg/mL) represent the NC and PC, respectively. All data are presented as the mean ± standard deviation (*n* = 3); the different letters (a–e) indicate statistical significance (*p* < 0.05). Significant differences were evaluated using the one-way analysis of variance followed by Duncan’s test for multiple comparisons. NC, negative control; PC, positive control.

Macrophages are primarily harvested from the bone marrow, spleen, and peritoneal cavity. Peritoneal macrophages are widely used because of their easy collection and stable phenotype. The immunostimulatory activity of the *Saccharina japonica* polysacchatride (SJP) on murine peritoneal macrophages was evaluated. Initially, the lipopolysaccharide (LPS), serving in the role of the positive control (PC) group, dramatically elevated the levels of IL-6, IL-12, and TNF-α released by macrophages (Figure 1). In addition, SJP stimulated the release of various cytokines (Figure 1a–c) from murine peritoneal macrophages. A notable observation was the concentration-dependent enhancement of TNF-α production by SJP, observed within the dose range of 1–1000 μg/mL. However, IL-6 and IL-12 exhibited a concentration-dependent increase only at 1–100 μg/mL and showed a decreasing trend at the highest concentration. SJP at a concentration of 1 µg/mL induced minimal secretion of all cytokines, exhibiting a limited effect. In contrast, SJP at a concentration of 100 µg/mL induced cytokine secretion levels comparable to those observed in the PC group treated with LPS. This finding suggests that SJP effectively stimulates the immune response of macrophages.

IL-6 is an inflammatory cytokine that exerts regulatory effects on the body. Macrophages are known to generate IL-6 early in the immune response, playing a critical role in its initiation [18]. Additionally, TNF-α is essential for triggering inflammatory responses as well as facilitating their resolution, serving as a key mediator in these processes, thus acting as a potent cytotoxic factor that induces additional cytokines, which ultimately results in the apoptosis of cancer cells. IL-12 is pivotal for activating NK cells and CTLs, ultimately enhancing their cytolytic activity against tumor cells. Subsequently, IL-12 induces the release of interferon-gamma, which stimulates the immune network. Therefore, IL-12 released by macrophages is involved in its anti-cancer effect. In the current study, SJP increased the immune response of peritoneal macrophages, suggesting the anti-cancer effects of *S. japonica* polysaccharides.

### 2.2. Effects of Polysaccharide from S. japonica for NK Cells and CTLs

**Figure 2:**
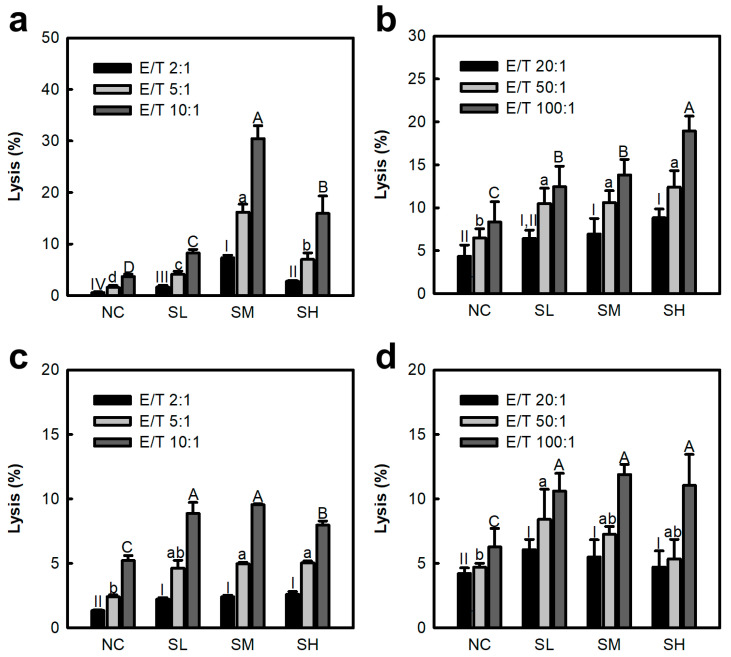
Comparison of NK cell and CTL cytotoxic activity against cancer cells by SJP. (**a**,**b**) Cytotoxicity of NK cells and CTLs by *i.v.* administration. (**c**,**d**) Cytotoxicity of NK cells and CTLs by oral administration. NK cells and CTLs were co-cultured with YAC-1 lymphoma and Colon26-M3.1 carcinoma, respectively, for 6 h in a 5% CO_2_ incubator at 37 °C. Saline and distilled water were administered to the NC groups. SJP was administered at various doses including at 0.5 (SL), 5 (SM), and 50 mg/kg (SH). All data are presented as the mean ± standard deviation (*n* = 3); the different letters (a–d, A–D, I–IV) indicate statistical significance (*p* < 0.05). Significant differences were evaluated using the one-way analysis of variance followed by Duncan’s test for multiple comparisons. NC, negative control; SL, SJP-low; SM, SJP-medium; SH, SJP-high.

NK cells and CTLs have potent functions in the removal of tumors and cancer cells. Previous studies have confirmed that SJP stimulates IL-12 secretion from peritoneal macrophages. The stimulatory function of IL-12 on effectors such as NK cells and CTLs has been verified in many studies. Therefore, the immunostimulatory effects of the polysaccharide fraction (SJP) isolated from *S. japonica* on cancer-cell-killing effects were assessed using splenic NK cells and CTLs. As depicted in Figure 2a,b, the intravenous (*i.v.*) administration of SJP increased the cytotoxicity of splenic NK cells and CTLs against YAC-1 lymphoma and Colon26-M3.1 carcinoma, respectively. In particular, the effect on NK cells was dose-dependent in the SL (0.5 mg/kg) and SM (5 mg/kg) groups; however, a slight decrease was observed in the SH (50 mg/kg) group (Figure 2a). This trend is consistent with previous results of IL-12 secretion by peritoneal macrophages (Figure 1b). However, a dose-dependent increase in cytotoxicity of CTLs was observed in the SL-SH group. The dissimilarity in the tendencies between NK cells and CTLs has been attributed to the intricate process of sensitization. The effects of oral administration were further investigated. As depicted in Figure 2c,d, oral administration of SJP was also found to improve cytotoxicity of NK cells and CTLs against cancer cells. In addition, NK-cell- and CTL-mediated cytotoxicity against tumor cells increased in an E/T-ratio-dependent manner across all results. These results suggest that the numbers of NK cells and CTLs have a significant effect on tumor-cell-killing effect. Therefore, SJP has been demonstrated to possess potent immunostimulatory effects in vivo.

NK cells make up 5–15% of the lymphocytes circulating in the blood. They accurately identify non-self and mutant genes based on the presence or absence of major histocompatibility complex class I molecules. Subsequently, NK cells destroy cancer cells via secreting cytotoxic enzymes [19]. As this process occurs, except for pre-sensitization through the antigen-presenting step, NK cells conduct a major mission in inhibiting early metastasis of cancer cells. In contrast, CTLs require prior sensitization by antigen-presenting cells. Their target destruction mechanisms are similar to those of NK cells in that they use granzyme and perforin. Moreover, *Coriolus versicolor* polysaccharide (Krestin), a commercially available immunostimulating anti-cancer drug, exhibits anti-cancer effects by NK cell and CTL activation [20]. These results confirm that SJP activates NK cells and CTLs, which are representative effectors of innate and adaptive immunity. These results suggest that SJP may contribute to anti-cancer effects by activating the entire immune system response.

### 2.3. Inhibitory Efficacy of SJP Against Lung Cancer

**Figure 3:**
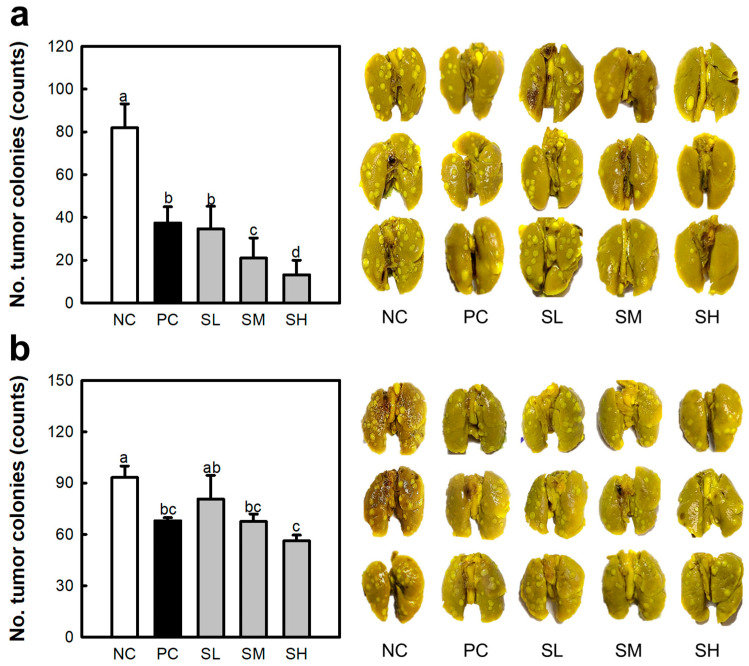
Inhibitory effects of SJP on Colon26-M3.1-caused lung cancer in BALB/c mice. (**a**) Intravenous administration. (**b**) Oral administration. Lung-cancer-bearing mice model induced by the *i.v.* inoculation of Colon26-M3.1 carcinoma. Saline and distilled water were administered to the NC group. Krestin (50 mg/kg), a known reference drug isolated from *Coriolus versicolor*, was used in the PC group. SJP was intravenously and orally administered at various doses including at 0.5 (SL), 5 (SM), and 50 mg/kg (SH), followed by *i.v.* inoculation with Colon26-M3.1 carcinoma. All data are presented as the mean ± standard deviation (*n* = 8); the different letters (a–d) indicate statistical significance (*p* < 0.05). Significant differences were evaluated using the one-way analysis of variance followed by Duncan’s test for multiple comparisons. NC, negative control; PC, positive control; SL, SJP-low; SM, SJP-medium; SH, SJP-high.

The anti-cancer effects of SJP were studied under two conditions: *i.v.* and oral administration. Lung-cancer-bearing mice were modeled using Colon26-M3.1 carcinoma. The NC group showed numerous tumor colonies (approximately 81) throughout the lung tissue (Figure 3a). However, *i.v.* administration of Krestin (PC group), an established standard drug obtained from *Coriolus versicolor*, potently inhibited the development of lung cancer (approximately 37). In addition, *i.v.* administration of SJP showed a dose-dependent inhibitory tendency with 34 (SL), 21 (SM), and 13 (SH) cancer cell colonies. In particular, 0.5 mg/kg SJP (SL group) showed similar effects to 50 mg/kg Krestin (PC group). At doses higher than 5 mg/kg, SJP demonstrated a more potent inhibitory effect than PC. The outcomes showed a stark contrast in colonies between the SH and NC groups. As shown in Figure 3b, oral administration of Krestin (approximately 68) showed notable inhibitory effects; however, this effect was weaker than that of *i.v.* administration of Krestin. In addition, all groups administered SJP showed a dose-dependent effect on cancer inhibition, which was most intense in the SH group (approximately 56). SJP demonstrated exceptional anti-cancer properties when administered orally or intravenously. According to the results of the oral administration, SJP demonstrated the ability to stimulate the immune response without being degraded by the digestive system. Moreover, SJP administration significantly affected both the number of tumor colonies and the reduction in their size. Briefly, the lungs of the NC group exhibited an irregular tissue surface due to tumor colonies, whereas the lungs of the SH group displayed a smooth and uniform morphology. These findings suggest that SJP exerts a potent therapeutic effect against lung cancer. Several natural polysaccharides have been reported to exhibit immune-enhancing properties that suppress tumorigenesis and metastasis [21,22]. Thus, stimulation of the immune response via polysaccharides is widely recognized as a crucial mechanism for combating cancer and metastasis. The present results demonstrate that SJP exhibits immune-enhancing and anti-cancer effects. However, the correlation between these two effects is insufficient. Therefore, further investigation of the correlation between the immune response and anti-cancer effects is needed.

### 2.4. Anti-Cancer Effects of SJP in NK Cell Function Depleted BALB/c Mice

**Figure 4:**
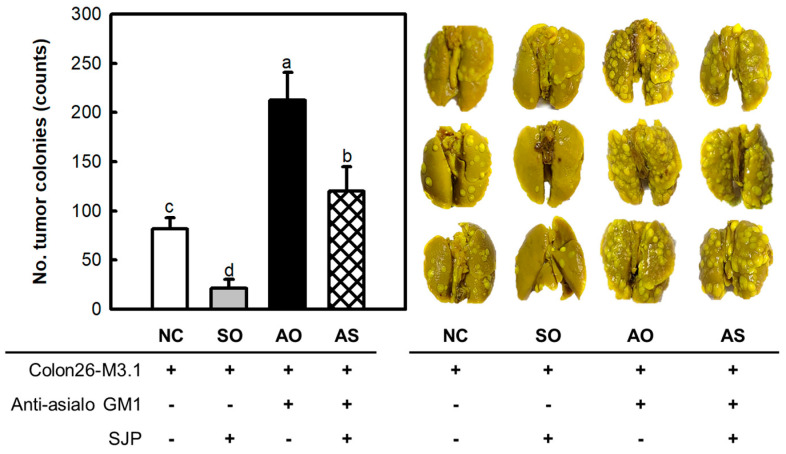
Cancer inhibitory effects of SJP on lung cancer in BALB/c mice with impaired NK cell function. Lung-cancer-bearing mice model induced by the *i.v.* inoculation of Colon26-M3.1 carcinoma. Saline was administered to the NC group. SJP was intravenously administered at 5 mg/kg, followed by *i.v.* inoculation with Colon26-M3.1 carcinoma. The rabbit anti-asialo-GM1 serum was intravenously injected into the mice, two days before inoculation with Colon26-M3.1 carcinoma, to deplete NK cell function. All data are presented as the mean ± standard deviation (*n* = 8); the different letters (a–d) indicate statistical significance (*p* < 0.05). Significant differences were evaluated using the one-way analysis of variance followed by Duncan’s test for multiple comparisons. NC, negative control; SO, SJP-only; AO, anti-asialo-GM1-only; AS, anti-asialo-GM1-SJP.

Earlier, SJP was shown to possess potent immunostimulatory and anti-cancer effects. The importance of immunity in achieving anti-cancer effects has been emphasized by numerous studies. Therefore, the influence of the immune response on the anti-cancer effects of SJP was investigated using additional animal models. These experimental models were characterized by the suppression of NK cell activity through the administration of anti-asialo-GM1. Briefly, asialo GM1 is a glycosphingolipid expressed on the surface of cells, and it is known to function as a receptor in antigen and pathogen invasion. The anti-asialo-GM1 antibody binds to asialo GM1, thereby inhibiting the antigen recognition ability of NK cells. On the other hand, the expression of asialo GM1 is observed on various immune cells, including NK cells, macrophages, and T cells. However, NK cells are reported to express asialo GM1 at relatively higher levels compared to other immune cells. In addition, other immune cells exhibit low levels of asialo GM1 expression, and depletion using anti-asialo-GM1 does not significantly impair the activity of other immune cells [23,24,25]. As depicted in Figure 4, inoculation of Colon26-M3.1 carcinoma in normal mice (NC group) induced colony development in the lung tissue. In addition, the SJP-only (SO) group showed significant anti-cancer effects, similar to previous results. Interestingly, the anti-asialo-GM1-only (AO) group showed an explosive increase in tumor colonies, which exceeded that of the NC group. These findings suggest a significant contribution of NK cells in anti-cancer responses. In addition, the anti-asialo-GM1-SJP (AS) group showed fewer tumor colonies than the AO group, despite the blockade of NK cell function. Therefore, the anti-cancer effects of SJP may be related to macrophages, NK cells, and CTLs.

Protecting the body from cancer initiation and metastasis, the immune system plays an indispensable role in maintaining defense mechanisms. To achieve the goal of overcoming cancer, a range of immunocytes which are critical for effective anti-cancer responses must be considered [26]. The presence of SJP in the presence (AS group) or absence (SO group) of anti-asialo-GM1 resulted in significant changes in cancer cell colonies, suggesting a profound involvement of NK cells in the anti-cancer effects of SJP. Similarly, comparisons between the AO and AS groups indicated that other factors were involved, in addition to NK cells. In brief, SJP did not exhibit direct cytotoxic effects against several tumor cells such as YAC-1 lymphoma, Colon26-M3.1 carcinoma, or B16BL6 melanoma. However, SJP showed a heightened level of cytotoxicity against NK cells and CTLs. Additionally, immune function of macrophages, representative initiators of the immune response, is stimulated in vitro. The findings indicate a strong connection between the anti-cancer activity by SJP and its ability to improve immune responses. In many studies, various factors influencing anti-cancer effects have been investigated, among which immune therapy is recognized as a well-established treatment for various types of cancer [27]. In immunotherapy, cytokine secretion and cytolytic activity by immune cells such as macrophages, NK cells, and CTLs play a crucial role. This study clearly indicated that SJP extracted from *S. japonica* triggered immune responses such as cytokine secretion and cytolytic effects and further inhibited the growth of lung cancer induced by inoculated cancerous cells. However, the molecular mechanisms underlying these activities are not yet fully understood and additional research is required to elucidate this aspect. This constitutes an important area that warrants further investigation.

### 2.5. Determination of Active Region of S. japonica

**Figure 5:**
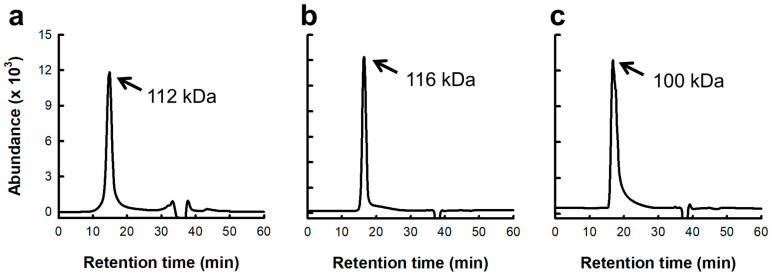
Molecular weight of polysaccharide from *S. japonica* on RID-HPLC. (**a**) SJP. (**b**) SJP-CS. (**c**) SJP-CP. Elution pattern of three polysaccharide fractions on RID-HPLC equipped with Superdex 75 column, stabilized with 50 mM ammonium formate buffer (pH 5.5).

Based on the above results, SJP showed potent immunostimulatory and anti-cancer effects. However, crude polysaccharides were not purified. Therefore, the sugar composition of SJP was primarily determined. As shown in Table 1, SJP mainly consists of seven different monosaccharides, ManA (59.7%), GulA (18.8%), Fuc (9.4%), glucuronic acid (GlcA, 4.4%), galactose (Gal, 4.8%), mannose (Man, 2.2%), and xylose (Xyl, 0.7%), which are typical sugar components of alginate and fucoidan. Moreover, the molecular weight distribution of SJP was detected as a symmetrical single peak, indicating that the molecular weights of the alginate and fucoidan fractions in SJP are quite similar (Figure 5a). The estimated molecular weight of SJP using the pullulan series was approximately 112 kDa.

**Figure 6:**
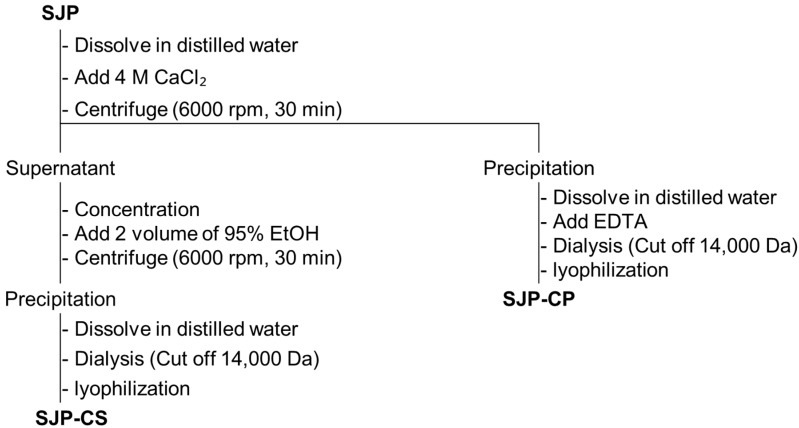
Scheme tree to separate polysaccharide from *S. japonica*.

**Figure 7:**
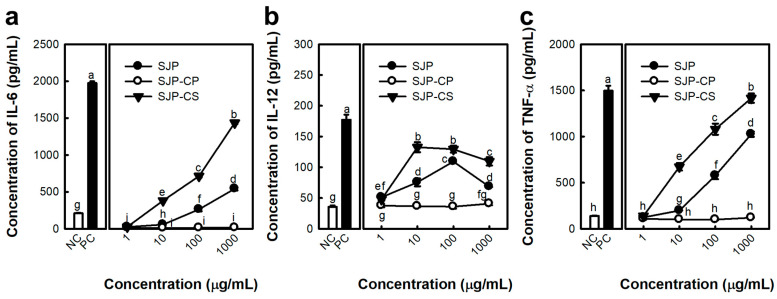
Effects of polysaccharide fractions isolated from *S. japonica* on cytokine secretion of murine peritoneal macrophages. (**a**) IL-6, (**b**) IL-12, and (**c**) TNF-α concentration of culture supernatant. The medium and LPS (1 μg/mL) represent the NC and PC, respectively. All data are presented as the mean ± standard deviation (*n* = 3); the different letters (a–i) indicate statistical significance (*p* < 0.05). Significant differences were evaluated using the one-way analysis of variance followed by Duncan’s test for multiple comparisons. Among the experimental groups, statistical significance was set at *p* value < 0.05. NC, negative control; PC, positive control.

In general, polysaccharides with high uronic acid content are precipitated by forming stable polymers in the presence of Ca^2+^ ions because of their carboxyl groups [28]. Therefore, the process presented in Figure 6 was used to separate the alginate fraction as a precipitate. Consequently, the alginate and fucoidan present in SJP were separated by the precipitation of uronic acid using CaCl_2_, and two fractions, SJP-CaCl_2_-Supernatant (SJP-CS) and SJP-CaCl_2_-Precipitate (SJP-CP), were obtained (Figure 6). First, the sugar composition of SJP-CS and SJP-CP was determined. SJP-CS mainly consisted of eight different monosaccharides: Fuc (51.7%), Gal (18.8%), GlcA (10.0%), Man (8.9%), ManA (5.9%), Xyl (2.1), GulA (1.5), and rhamnose (Rha, 1.0%). Although small amounts of alginate-related monosaccharides, such as GulA and ManA, were present, most of the monosaccharides matched the possible sugar composition of fucoidan. In contrast, the sugar component of SJP-CP was monotonous. Briefly, ManA and GulA accounted for 73.0% and 21.9% of the SJP-CP, respectively. The sugar components of the two fractions suggested that the separation process with CaCl_2_ was suitable. Furthermore, the molecular weight distribution results for SJP-CS (Figure 5b) and SJP-CP (Figure 5c) indicated their high purity. The molecular weights of SJP-CS and SJP-CP were calculated to be 116 and 100 kDa, respectively. The effect of each fraction on macrophage activation was notable. SJP-CS demonstrated a concentration-dependent effect on cytokine secretion, which was statistically significant when compared with SJP (Figure 7). In contrast, SJP-CP did not induce any cytokines from peritoneal macrophages. The above results helped us understand the active moiety present in SJP. SJP was discovered to be a combination of alginate (SJP-CP) and fucoidan (SJP-CS) with comparable molecular weights and was successfully isolated using CaCl_2_. SJP-CP, the alginate fraction, did not exhibit any immunostimulatory effects, whereas SJP-CS, the fucoidan fraction, demonstrated a more significant immunostimulatory effect when compared to SJP. These results suggested that the active moiety of the polysaccharide fraction from *S. japonica* is fucoidan.

## 3. Materials and Methods

### 3.1. Materials and Animals

The polysaccharide fraction of *S. japonica* was isolated through several steps. Dried *S. japonica* powder was subjected to hot water extraction (80 °C, 5 h), and the residue was subsequently discarded by centrifuge. The polysaccharide fraction was specifically obtained using ethanol precipitation (95% ethanol, addition volume: ×1.5), dialysis, and lyophilization. After that, the dried powder was named *Saccharina japonica* polysaccharide (SJP).

Female SPF BALB/c mice, aged 6 weeks, were used to confirm anti-cancer effects of polysaccharide. They were kept in sterilized cages under conditions of 23 °C, 55% humidity, and a 12 h light/dark cycle. Food pellets and water were provided without restriction, ensuring ad libitum access.

### 3.2. Cytokine Release from Peritoneal Macrophages of Mice

Macrophages were induced in the peritoneum using matured 5% thioglycolate (TG) for 3 months [29,30]. In short, 5% TG (1 mL) was administered intraperitoneally into mice. After 96 h, macrophages were harvested using phosphate-buffered saline. The collected macrophages (2.0 × 10^5^ cells/well) were plated in a 96-well microplate. Thereafter, various concentrations of samples were added and left for 24 h in a CO_2_ incubator (37 °C). Medium and LPS were used as NC and PC groups, respectively. LPS was added under the same conditions as the sample, at a concentration of 1 µg/mL. Subsequently, culture supernatant was used for sandwich ELISA for cytokines. ELISA kits for IL-6 and IL-12 quantification secreted in culture supernatant were purchased from BD Bioscience (San Diego, CA, USA). ELISA kits for TNF-α analysis were purchased from Invitrogen (San Diego, CA, USA). All ELISA kits were utilized in accordance with the protocols provided by the manufacturer.

### 3.3. NK-Cell-Related Cytolytic Effect

The effects of the polysaccharides on the cytolytic activity of NK cells against cancer cells were investigated. In brief, SJP was administered at various doses, including 0.5 (SL), 5 (SM), and 50 mg/kg (SH). The samples were provided through *i.v.* on days 1 and 3 before euthanasia or administered orally once a day for 28 consecutive days prior to euthanasia. Saline (*i.v.*) and distilled water (oral) were orally administered to the NC group. NK cells were selectively harvested using a mouse NK cell isolation kit. YAC-1 lymphoma cells, characterized by their lack of MHC-I expression, are frequently employed as target cells in studies of NK cell functionality. YAC-1 lymphoma (target) was seeded at a 5.0 × 10^4^ cells/well in a 96-well microplate. The NK cells (effector) were cultured together with target cells at effector-to-target (E/T) ratios of 10:1, 5:1, and 2:1. The cytotoxic activity of NK cells was assessed by quantifying the release of lactate dehydrogenase (LDH) into the culture supernatant, which occurred as a result of YAC-1 lymphoma cell lysis. LDH levels were quantified by the EZ-LDH assay kit.

### 3.4. Cytotoxic-T-Lymphocyte-Mediated Cytolytic Effect on Colon26-M3.1

The effects of the polysaccharides on the cytolytic activity of CTLs against cancer cells were measured. In brief, SJP was administered with various doses, including 0.5 (SL), 5 (SM), and 50 mg/kg (SH). The samples were provided through *i.v.* on days 1 and 3 before euthanasia or administered orally once a day for 28 consecutive days prior to euthanasia. Saline (*i.v.*) and distilled water (oral) were orally administered to the NC group. Subsequently, the CTLs were pre-sensitized by inoculating with a Colon26-M3.1 carcinoma (target). CTLs were selectively harvested using a CD8a^+^ cell isolation kit. Colon26-M3.1 carcinoma (target) was seeded at 1.0 × 10^4^ cells/well in a 96-well microplate. The CTLs (effector) were co-seeded with target at ratios of E/T set to 100:1, 50:1, and 20:1. Subsequent procedures for determining LDH levels were identical to those described in Section 3.3.

### 3.5. Lung-Cancer-Bearing Mice Model

The therapeutic potential of SJP against lung cancer was assessed in a mouse model developed with Colon26-M3.1. Briefly, BALB/c mice were intravenously injected with Colon26-M3.1 (3 × 10^4^) to establish the model. The *i.v.* administration of SJP was performed on days 1 and 3 before the inoculation, while oral administration was conducted daily for 28 consecutive days leading up to the inoculation. Krestin isolated from *Coriolus versicolor*, an immunostimulatory anti-cancer reference drug, was used as the PC group [31]. Fourteen days after cancer cell inoculation, all experimental mice were euthanized. Following this, all lung tissues were preserved using Bouin’s solution, and the colonies were examined and quantified under a microscope.

### 3.6. NK-Cell-Activity-Impaired Mice Model

NK-cell-function-deficient mice were generated by administering anti-asialo-GM1 antibody to inhibit NK cell activity [24]. Briefly, 5 mg/kg SJP was intravenously administered to BALB/c two times, specifically on days 1 and 3 prior to the inoculation of cancer cells. Moreover, anti-asialo-GM1 was intravenously administered to BALB/c mice two days before cancer cell inoculation. Moreover, BALB/c mice were intravenously injected with Colon26-M3.1 (3 × 10^4^) to establish the model. After that, the subsequent procedure was carried out in the same manner as described in Section 3.5.

### 3.7. Fractionation of Polysaccharide

To determine the active moiety of SJP, fractionation was performed using CaCl_2_. Briefly, SJP (10 g) was dissolved using water, and 4 M CaCl_2_ (1 L) was added to separate the fucoidan and alginate fractions. The supernatant fraction by CaCl_2_ was further fractionated using 95% ethanol precipitation and centrifugation (6000 rpm, 30 min). Finally, the precipitated fraction was dialyzed (molecular weight cut-off: 13,000 Da) and named SJP-CaCl_2_-Superntant (SJP-CS). The precipitated fraction of CaCl_2_ was dissolved using water and EDTA. The result was dialyzed (molecular weight cut-off: 13,000 Da) and named SJP-CaCl_2_-Precipitate (SJP-CP).

### 3.8. Component Sugar

The component sugar was investigated as described following the previous study [32]. In brief, polysaccharide fractions were hydrolyzed using 2 M trifluoroacetic acid at 121 °C for 90 min. Subsequently, the result was reacted with 1-phenyl-3-methyl-5-pyrazolone (PMP) under base conditions at 70 °C for 100 min. The final derivatized samples were determined using a UVD-HPLC system (LC-20AD, Shimadzu Ltd., Kyoto, Japan) equipped with a C18 column (Acclaim^TM^ 120, Thermo Fisher, Waltham, MA, USA). The mobile phase was composed of 0.05 M sodium phosphate buffer (pH 6.7) and acetonitrile in an 82:18 (*v*/*v*) ratio. The experiments were performed under isocratic conditions. The analysis was conducted at a flow rate of 1 mL/min for 60 min, with the column oven temperature maintained at 30 °C.

### 3.9. Molecular Weight

Molecular weight of the samples was analyzed using the RID-HPLC system (Agilent 1260 Infinity; Agilent Technologies, Palo Alto, CA, USA) equipped with a Superdex 75 column (GE Healthcare, Piscataway, NJ, USA). The mobile phase was composed of 0.05 M ammonium formate buffer (pH 5.5). The experiments were conducted under isocratic conditions. The analysis was carried out at a flow rate of 0.5 mL/min for a duration of 60 min, with the column oven temperature consistently maintained at 30 °C. Through the pullulan series, the molecular weight was determined.

### 3.10. Statistical Test

All statistical analyses were carried out with SPSS version 21.0. All data are expressed as the mean ± standard deviation. Group differences were analyzed through one-way ANOVA, followed by Duncan’s multiple comparison test, with statistical significance defined as *p* < 0.05.

## 4. Conclusions

This study investigated the immunostimulatory effects of a polysaccharide fraction (SJP) derived from *S. japonica*. SJP activates various effectors of the immune system, including macrophages, NK cells, and CTLs, both in vitro and in vivo. Consistently, these effects are considered to correlate with anti-cancer effects. Furthermore, fractionation of SJP was accomplished using CaCl_2_, which verified that fucoidan was the active moiety of the polysaccharide fraction derived from *S. japonica*. In many studies, various factors influencing anti-cancer effects have been investigated, among which immune therapy is recognized as a well-established treatment for various types of cancer. In this study, the correlation between the anti-cancer effects and immune responses to *S. japonica* polysaccharides was preliminarily addressed. Accurate structural information of the active moiety is essential for understanding the molecular mechanisms underlying these biological effects. Mass spectrometry and nuclear magnetic resonance can be used to analyze the structure of polysaccharides. Our goal was to identify the structural features and molecular mechanisms involved in the immunostimulatory effects of the fucoidan fraction.

## Figures and Tables

**Table 1:** Sugar composition of polysaccharide fractions isolated from *S. japonica*.

Component Sugar (Mole %)	SJP	SJP-CS *	SJP-CP **
Rhamnose	-	1.0 ± 0.0	-
Fucose	9.4 ± 0.3	51.7 ± 0.2	3.4 ± 0.5
Arabinose	-	-	-
Xylose	0.7 ± 0.0	2.1 ± 0.1	-
Mannose	2.2 ± 0.1	8.9 ± 0.1	1.7 ± 0.2
Galactose	4.8 ± 0.1	18.8 ± 0.0	-
Glucose	-	-	-
Glucuronic acid	4.4 ± 0.2	10.0 ± 0.3	-
Galacturonic acid	-	-	-
Guluronic acid	18.8 ± 1.6	1.5 ± 0.2	21.9 ± 0.9
Mannuronic acid	59.7 ± 2.0	5.9 ± 0.2	73.0 ± 1.7

* SJP-CS: SJP-CaCl_2_-Superntant; ** SJP-CP: SJP-CaCl_2_-Precipitate.

## Data Availability

Data will be made available on request.
